# Diagnostic accuracy of smartphone and macro camera imaging for dental caries and oral health conditions

**DOI:** 10.1186/s12903-026-08523-z

**Published:** 2026-05-01

**Authors:** Beyza Cakmakci, Gul Yildiz Telatar

**Affiliations:** 1 Department of Restorative Dentistry, Kocaeli Derince Oral And Dental Health Center, Kocaeli, Turkey; 2 Department of Restorative Dentistry, Burdur Mehmet Akif Ersoy University, Faculty of Dentistry, Burdur, Turkey

**Keywords:** Oral health, Smartphone, Telemedicine, Dental caries, Oral hygiene parameters

## Abstract

**Background:**

This study aimed to assess the diagnostic accuracy of digital intraoral photographs obtained using smartphones and a macro camera in evaluating oral health among adults.

**Methods:**

A total of 200 adult patients underwent clinical and radiographic examinations using the Decayed, Filled Teeth (DFT) Index, Caries Assessment Spectrum and Treatment (CAST) Index, Plaque Index (PI), and Modified Gingival Index (MGI). Intraoral photographs were taken using three devices: Samsung S23 Ultra, iPhone 14 Pro, and Canon EOS 400D with macro lens. Following the clinical recording of DFT, CAST, PI, and MGI scores by two calibrated examiners as the reference standard, intraoral photographs were captured by a third dentist and independently evaluated by two separate blinded examiners to compare the diagnostic accuracy of the devices against the clinical findings. Non-parametric analyses were conducted using the Friedman test with Dunn’s post hoc test, Wilcoxon test and agreement between clinical and photographic methods was evaluated via the Bland–Altman method (*p* < 0.05).

**Results:**

The macro camera demonstrated the highest inter-rater reliability for FT scores (ICC = 0.886), while iPhone-derived MGI scores showed the lowest reliability (ICC = 0.624). Statistically significant differences were found among all imaging devices for all indices (*p* < 0.001), except for MGI. Bland–Altman analysis showed that most values fell within the 95% limits of agreement, indicating good concordance with clinical data.

**Conclusions:**

Smartphone and macro camera photographs provided comparable diagnostic results for caries and restorations. However, limitations remain in the assessment of periodontal parameters via photographic methods. Smartphone-based intraoral photography can serve as a practical diagnostic tool in teledentistry.

## Background

Teledentistry is a branch of telehealth that leverages digital technology and telecommunications to support the diagnosis, treatment, consultation, education, and public awareness of oral and dental health [1, 2]. With the growing adoption of digital platforms, teledentistry has become an increasingly common and practical alternative for screening oral diseases and dental conditions [3].

Teledentistry facilitates the early detection and management of soft tissue lesions, the prevention of dental caries, and the implementation of less invasive interventions that can improve clinical outcomes [4]. In addition, teledentistry enables long-term monitoring and follow-up through digital archiving and the use of standardized indices [5–7]. Numerous studies have demonstrated the feasibility of evaluating oral health status using intraoral digital photographs [8–10]. Dental caries, a widespread chronic infectious disease, does not resolve spontaneously but can be managed through effective oral hygiene practices and regular dental checkups [11]. In-person examinations require clinical infrastructure, trained personnel, patient travel, and chairside time, all of which contribute to increased costs and logistical burden [12]. Intraoral photographs taken by healthcare personnel using smartphones have been shown to support reliable assessments of plaque accumulation and gingival health when reviewed by trained dental professionals [13].

By overcoming barriers such as geographic isolation, socioeconomic limitations, and shortages of dental professionals, teledentistry offers a promising solution for expanding access to early caries detection and oral disease management [14]. Recent systematic reviews have demonstrated that smartphone-based and photographic methods provide reliable diagnostic performance for dental caries detection, with results comparable to clinical examinations. Furthermore, photographic assessment has shown promising accuracy in evaluating plaque accumulation and gingival conditions, although variability exists depending on image quality and clinical parameters. These findings highlight the growing role of digital imaging in teledentistry and emphasize the need for further studies evaluating multiple indices simultaneously [15–17].

Although previous studies have evaluated teledentistry using individual indices or specific imaging modalities, limited research has comprehensively assessed multiple indices such as the Decayed, Filled Teeth (DFT) Index, Caries Assessment Spectrum and Treatment (CAST) Index, Plaque Index (PI), and Modified Gingival Index (MGI) simultaneously across different imaging devices. The present study aims to evaluate the accuracy and efficiency of intraoral digital photography in remote oral health assessments, hypothesizing that this method may offer diagnostic performance comparable to conventional clinical examinations.

## Methods

### Ethics approval

This study adhered to the Strengthening the Reporting of Observational studies in Epidemiology (STROBE) guidelines [18]. Ethical approval for the study was obtained from the Non-Invasive Clinical Research Ethics Committee of Recep Tayyip Erdoğan University Faculty of Medicine (approval number: 2023/115). A signed and informed constent form was obtained from all volunteers for cooperation to use digital photographs taken.

### Eligibility criteria

Inclusion criteria included individuals aged 18–64 years with no systemic or psychiatric conditions that could interfere with examination procedures. Exclusion criteria included individuals with severe systemic diseases, uncooperative behavior, or conditions preventing adequate intraoral imaging.These conditions were excluded to ensure participant cooperation, reliability of clinical assessments, and standardization of image acquisition.

### Sample size

In the study, the sample size was calculated at a 95% confidence level using the “G. Power-3.1.9.2” program. As a result of the analysis, α = 0.05, the standardized effect size was calculated as 0.664 from the previous study [12], and the minimum sample size per group was obtained as 37 with a theoretical power of 0.80. To address data losses, it was decided to engage a total of 200 individuals, divided into groups of 50. The calculated sample size referred to the minimum number of subjects required to detect differences between repeated measurements obtained from different imaging modalities (iPhone, Samsung, macro camera, and clinical examination), rather than independent study groups.

### Clinical examination and image protocol

**Fig. 1:**
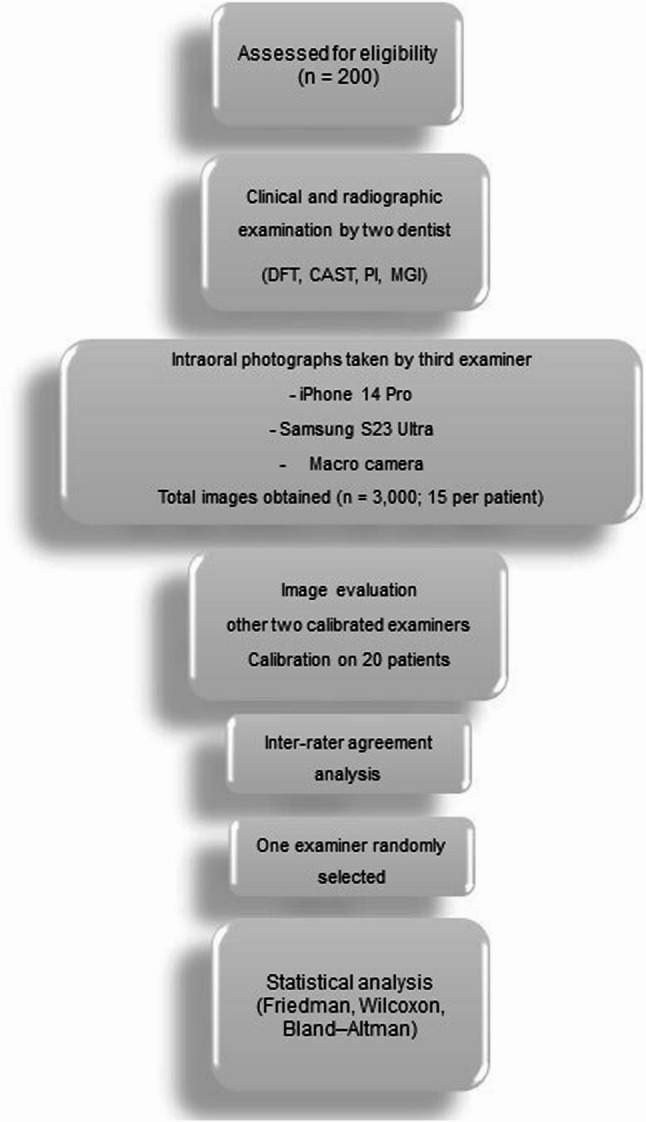
Flowchart of the diagnostic workflow comparing clinical examination and digital imaging groups

Flowchart of the diagnostic workflow comparing clinical examination and digital imaging groups was presented in Fig. 1.

The study was conducted at the Department of Restorative Dentistry, Faculty of Dentistry, Recep Tayyip Erdogan University, between December 2023 and October 2024.

To minimize bias and ensure measurement reliability, all procedures were performed in accordance with a strict blinding protocol. Clinical examinations were conducted by two dentists calibrated to standardized indices to record DFT, CAST, PI, and MGI scores.

Intraoral photographs were obtained using various devices by a single dentist experienced in dental photography who was not involved in the clinical or photographic evaluations.

For the photographic assessment phase, other two calibrated examiners independently evaluated the images while blinded to clinical and photographic examination.

Patients attending the dental clinic who provided informed consent were examined by two calibrated dentists. Calibration was performed on 20 patients who were not included in the main study population, using the DFT, CAST, PI, and MGI. Clinical assessments comprised both intraoral examinations and radiographic evaluations, including panoramic radiographs and, when necessary, supplemental bitewing or periapical radiographs. Extracted teeth and third molars were excluded from all analyses.

Following the clinical oral examination, intraoral photographs were obtained during the same session by a dentist experienced in dental photography. Digital images were captured using two smartphones (Samsung Galaxy S23 Ultra and iPhone 14 Pro) and a digital macro camera (Canon EOS 400D).

For smartphone imaging, photographs were taken without flash under standardized ambient lighting conditions (28 W matte white LED). Autofocus was achieved by tapping the screen, while digital zoom and HDR mode were disabled. Images were captured at a distance of approximately 10 cm, using lip–cheek retractors and positioning the camera perpendicular to the tooth surface.

Macro images were obtained using a Canon EOS 400D camera equipped with a 100 mm macro lens and a Macro Ring Lite MR-14EX II ring flash. The camera was operated in manual mode with standardized settings: aperture f/22, ISO 200, shutter speed 1/160 s, white balance set to “flash,” and flash output adjusted to 1/8 power. The shooting distance was approximately 20 cm, and the 100 mm macro lens allowed handheld image acquisition without the need for a tripod.

**Fig. 2:**
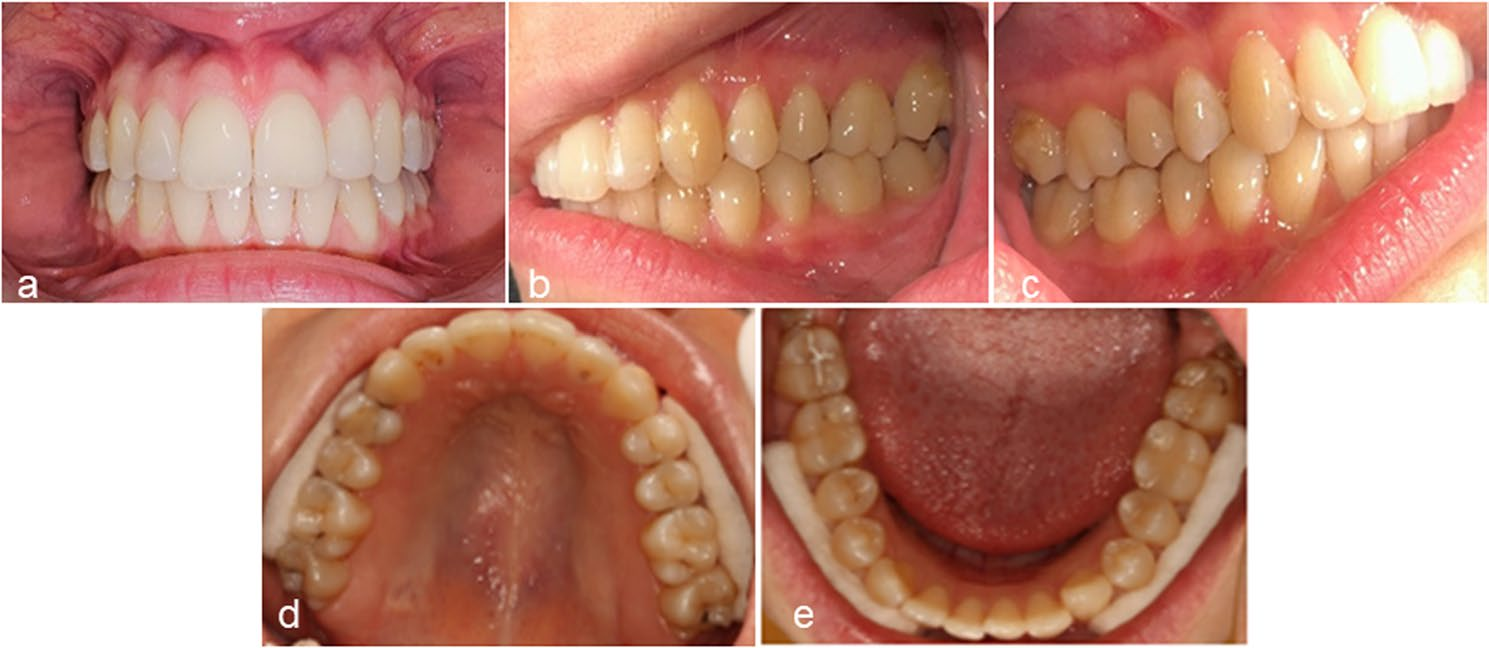
Sample Photographic Protocol (**a**) Anterior view, Samsung S23 Ultra; (**b**) Right lateral mirror view, iPhone 14 Pro; (**c**) Left lateral mirror view, iPhone 14 Pro; (**d**) Upper occlusal mirror view, macro camera; (**e**) Lower occlusal mirror view, macro camera

As illustrated in Fig. 2, five standardized photographs were taken per patient: one frontal view, two lateral views (right and left), and two occlusal views (upper and lower). This protocol yielded a total of 15 images per patient across the three imaging devices [12–14, 19]. Photographic procedures were conducted in accordance with previously published protocols [19]. All images were stored on a secure computer in folders labeled according to the imaging device used, to facilitate the digital evaluation of intraoral photographs. Two dentists, calibrated prior to the study using a separate group of 20 patients, independently assessed the photographs in a blinded manner. Both evaluators were unaware of the folder labels and used the same indices applied during the clinical examinations.

### Examination parameters

The PI and MGI were assessed for each participant according to standardized criteria. The PI was evaluated using the Silness and Löe method, with scores assigned as follows: 0 indicating no visible plaque; 1 representing a thin plaque layer along the gingival margin; 2 corresponding to a visible plaque in the gingival pocket and gingival margin; and 3 indicating a dense plaque in the gingival pocket and on the gingival margin. The MGI was recorded using a five-point scale: 0 denoted the absence of inflammation; 1 represented mild inflammation with slight changes in gingival color or texture; 2 indicated mild inflammation involving the entire gingival margin; 3 reflected moderate inflammation characterized by glossy redness, swelling, or gingival enlargement; and 4 denoted severe inflammation, including pronounced redness or hyperplasia, spontaneous bleeding, or ulceration [19]. The DFT was utilized to record both decayed teeth and those restored due to caries, in accordance with diagnostic criteria established by the World Health Organization [20]. The CAST is classified as follows: Code 0 indicates sound teeth with no visible signs of caries; Code 1 refers to the presence of sealants, either partial or complete, in pits and fissures; Code 2 denotes restorations involving direct or indirect restorative materials; Code 3 corresponds to enamel lesions, characterized by distinct color changes with or without loss of surface integrity; Code 4 indicates dentin lesions with color changes suggesting caries progression into the dentin, with or without enamel breakdown; Code 5 represents cavitated dentin lesions without pulp involvement; Code 6 denotes advanced caries with pulp exposure or remaining root fragments; Code 7 includes abscesses or fistulas related to pulpal infection; Code 8 refers to tooth loss due to caries; and Code 9 is assigned to other conditions not covered by the preceding categories. Notably, the “Missing” (M) component of the DMFT Index and Code 8 of the CAST Index—both representing tooth loss due to caries—were excluded from the analysis, as dental history could not be reliably determined from photographic records [15, 16, 21].

### Statistical methods

Cohen’s Kappa statistic was calculated to assess the agreement and consistency of index scores for 20 patients not included in the main study. Analyses were performed using IBM SPSS Statistics for Windows, Version 25 (IBM Corp., Armonk, NY, USA). The Kappa statistics for the first and second measurements across study groups ranged from 0.664 to 0.983, indicating substantial to almost perfect agreement. The inter-rater reliability exceeded the minimum threshold of 0.610, with statistically significant and high concordance (*p* < 0.05).

Data from the photographic evaluations were analyzed using IBM SPSS version 23 and Jamovi software. Normality of the data was assessed using the Kolmogorov-Smirnov test., The Friedman test was applied, followed by Dunn’s test for multiple comparisons. The Wilcoxon Signed-Rank Test was performed to compare each digital imaging modality individually against the clinical examination (gold standard). Agreement between the gold standard (clinical examination) and photographic assessments was evaluated using the Bland-Altman method. Graphs were generated using the “blandr” package in Jamovi. Results for quantitative variables were presented as mean ± standard deviation and median (minimum–maximum). Statistical significance was set at *p* < 0.05.

## Results

A total of 3,000 photographs were obtained from 200 patients, with 15 images captured per patient (5 per device). Inter-rater agreement analysis for the photographs taken using the three device types (iPhone, Samsung, and macro camera) is summarized in Table 1. High levels of statistical agreement were observed among raters for the DFT, FT, CAST, and PI parameters across all devices (< 0.001). Moderate agreement was noted for the DT and MGI parameters, regardless of the device used (< 0.001). Moderate agreement was observed for DT and MGI across all imaging modalities, whereas other parameters demonstrated good to excellent agreement. Based on these inter-rater reliability results, the observations of a single rater were randomly selected for subsequent analyses. Comparisons with the gold standard (clinical examination) were performed using data from this selected rater.

**Table 1:** Inter-rater agreement analysis

	IPHONE		SAMSUNG		MACRO	
	ICC (%95 CI)	*p*	ICC (%95 CI)	*p*	ICC (%95 CI)	*p*
DFT	0.787 (0.728–0.835)	**< 0.001**	0.837 (0.79–0.874)	**< 0.001**	0.857 (0.815–0.89)	**< 0.001**
DT	0.542 (0.436–0.633)	**< 0.001**	0.596 (0.498–0.678)	**< 0.001**	0.675 (0.592–0.744)	**< 0.001**
FT	0.831 (0.782–0.869)	**< 0.001**	0.862 (0.822–0.894)	**< 0.001**	0.886 (0.852–0.912)	**< 0.001**
CAST	0.786 (0.727–0.834)	**< 0.001**	0.800 (0.744–0.845)	**< 0.001**	0.796 (0.739–0.842)	**< 0.001**
PI	0.781 (0.721–0.83)	**< 0.001**	0.791 (0.733–0.838)	**< 0.001**	0.854 (0.812–0.888)	**< 0.001**
MGI	0.624 (0.531–0.702)	**< 0.001**	0.632 (0.54–0.708)	**< 0.001**	0.728 (0.656–0.787)	**< 0.001**

*ICC* Intraclass correlation coefficient, *CI* Confidence interval, *DFT* Decayed. Filled Teeth, *DT* Decayed Teeth, *FT* Filled Teeth, *CAST* Caries Assessment Spectrum and Treatment index, *PI* Plaque Index, *MGI* Modified Gingival Index; Bold values indicate statistically significant differences p < 0.05

The comparison of device performance is presented in Table 2. According to the results, statistically significant differences were observed among the evaluations based on photographs taken with the iPhone, Samsung smartphone, and macro camera for all assessed parameters, except for the MGI (< 0.001). The median DFT score obtained from the iPhone was 6, while the Samsung device also yielded a median DFT score of 6. In contrast, the macro camera produced a higher median DFT score of 7. Analysis of multiple comparisons revealed that the highest values were consistently associated with data obtained from the macro camera, whereas the lowest values were recorded with the iPhone.

**Table 2:** Comparison of measurements obtained from devices

	IPHONE		SAMSUNG		MACRO		p*
	mean ± sd	Median (min-max)	mean ± sd	Median (min-max)	mean ± sd	Median (min-max)	
DFT	6.3 ± 3.48	6 (0–20)^c^	6.77 ± 3.46	6 (0–16)^b^	7.13 ± 3.56	7 (0–17)^a^	**< 0.001**
DT	0.64 ± 0.97	0 (0–5)^b^	0.79 ± 1.12	0 (0–5)^ab^	0.96 ± 1.3	1 (0–7)^a^	**< 0.001**
FT	5.66 ± 3.55	5 (0–20)^b^	5.98 ± 3.53	5.5 (0–15)^a^	6.17 ± 3.65	6 (0–17)^a^	**< 0.001**
CAST	3.54 ± 1.16	3 (2–6)	3.61 ± 1.19	3 (2–6)	3.7 ± 1.14	4 (2–6)	**< 0.001**
PI	0.2 ± 0.34	0 (0–2.5)^b^	0.22 ± 0.36	0 (0–2.5)^ab^	0.24 ± 0.38	0.02 (0–2.5)^a^	**< 0.001**
MGI	0.15 ± 0.33	0 (0–2)	0.16 ± 0.36	0 (0–2)	0.17 ± 0.36	0 (0–2)	**0.001**

*Friedman test; ^a−c^: There is no difference between measurements with the same letter; Bold values indicate statistically significant differences p < 0.05

*SD* Standard deviation, *DFT* Decayed. Filled Teeth, *DT* Decayed Teeth, *FT* Filled Teeth, *CAST* Caries Assessment Spectrum and Treatment index, *PI* Plaque Index, *MGI* Modified Gingival Index

Comparison of digital imaging modalities with the clinical gold standard revealed statistically significant differences across all indices, including DFT, DT, FT, CAST, PI, and MGI (< 0.001). Clinical examination consistently yielded higher mean values than all digital devices, indicating a general trend of underestimation by teledentistry tools. Among the digital groups, the Macro camera demonstrated the closest mean values to the clinical gold standard across all evaluated parameters (Table 3).

**Table 3:** Comparison of device measurements with clinical examination

	CLINIC		IPHONE		SAMSUNG		MACRO		p*
	mean ± sd	Median (min-max)	mean ± sd	Median (min-max)	mean ± sd	Median (min-max)	mean ± sd	Median (min-max)	
DFT	8.75 ± 4.1	8 (1–26)	6.3 ± 3.48	6 (0–20)	6.77 ± 3.46	6 (0–16)	7.13 ± 3.56	7 (0–17)	**< 0.001**
DT	1.59 ± 2.01	1 (0–12)	0.64 ± 0.97	0 (0–5)	0.79 ± 1.12	0 (0–5)	0.96 ± 1.3	1 (0–7)	**< 0.001**
FT	7.16 ± 3.79	7 (0–18)	5.66 ± 3.55	5 (0–20)	5.98 ± 3.53	5.5 (0–15)	6.17 ± 3.65	6 (0–17)	**< 0.001**
CAST	4.23 ± 1.49	4 (2–7)	3.54 ± 1.16	3 (2–6)	3.61 ± 1.19	3 (2–6)	3.7 ± 1.14	4 (2–6)	**< 0.001**
PI	0.39 ± 0.49	0.16 (0–2.54)	0.2 ± 0.34	0 (0–2.5)	0.22 ± 0.36	0 (0–2.5)	0.24 ± 0.38	0.02 (0–2.5)	**< 0.001**
MGI	0.4 ± 0.6	0.07 (0–2.4)	0.15 ± 0.33	0 (0–2)	0.16 ± 0.36	0 (0–2)	0.17 ± 0.36	0 (0–2)	**< 0.001**

*Wilcoxon test; *SD* Standard deviation, *DFT* Decayed. Filled Teeth, *DT* Decayed Teeth, *FT* Filled Teeth, *CAST* Caries Assessment Spectrum and Treatment index, *PI* Plaque Index, *MGI* Modified Gingival Index; Bold values indicate statistically significant differences p < 0.05

The Bland-Altman plot facilitates visual examination of the differences between two measurement methods and their mean values. Differences between iPhone, Samsung, macro camera, and clinical evaluations for each parameter are presented using Bland-Altman plots. The lower and upper limits of agreement for the Bland-Altman plots are shown in Table 4.

**Table 4:** Lower and upper limits of agreement for Bland-Altman plots comparing iPhone. Samsung. and macro camera measurements with clinical measurements

Index	Comparison	Mean Difference (Bias)	Lower Limit of Agreement	Upper Limit of Agreement
DFT	IPHONE-CLINIC	-2.45	-3.090	7.990
	SAMSUNG-CLINIC	-1.98	-2.720	6.680
	MACRO-CLINIC	-1.62	-2.480	5.720
DT	IPHONE-CLINIC	-0.95	-2.30	4.190
	SAMSUNG-CLINIC	-0.80	-2.269	3.879
	MACRO-CLINIC	-0.63	-1.936	3.206
FT	IPHONE-CLINIC	-1.50	-3.230	6.230
	SAMSUNG-CLINIC	-1.18	-2.730	5.080
	MACRO-CLINIC	-0.99	-2.466	4.436
CAST	IPHONE-CLINIC	-0.69	-1.540	2.910
	SAMSUNG-CLINIC	-0.62	-1.597	2.827
	MACRO-CLINIC	-0.53	-1.744	2.804
PI	IPHONE-CLINIC	-0.19	-0.350	0.730
	SAMSUNG-CLINIC	-0.17	-0.330	0.670
	MACRO-CLINIC	-0.15	-0.348	0.636
MGI	IPHONE-CLINIC	-0.25	-0.630	1.130
	SAMSUNG-CLINIC	-0.24	-0.603)	1.082
	MACRO-CLINIC	-0.23	-0.598	1.061

*DFT* Decayed. Filled Teeth, *DT* Decayed Teeth, *FT* Filled Teeth, *CAST* Caries Assessment Spectrum and Treatment index, *PI* Plaque Index, *MGI* Modified Gingival Index

**Fig. 3:**
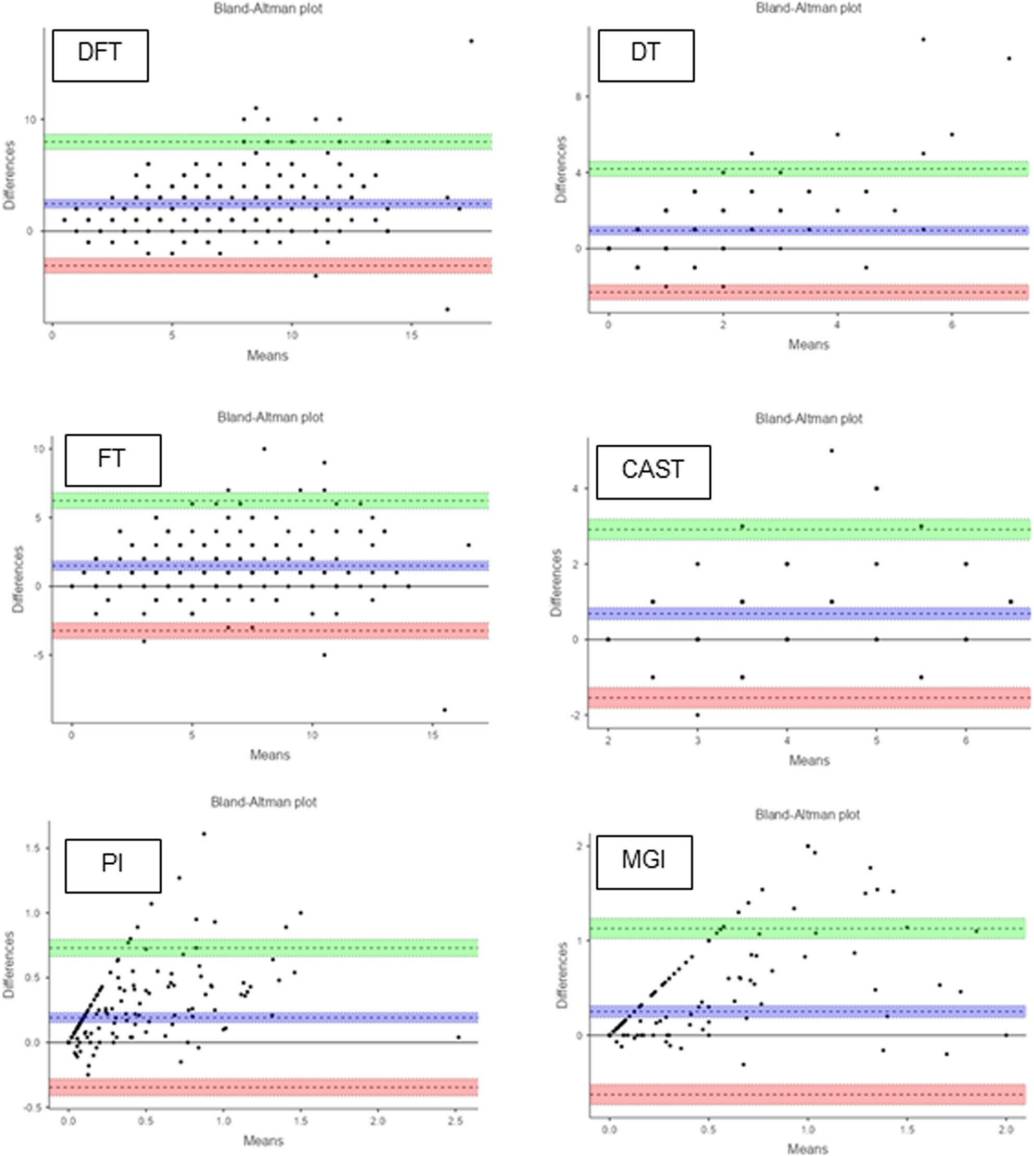
Bland-Altman plots for index measurements comparing IPhone and clinical examination (**a**) DFT Index; (**b**) DT Index; (**c**) FT Index; (**d**) CAST Index; (**e**) PI; (**f**) MGI. DFT, Decayed, Filled Teeth; DT, Decayed Teeth; FT, Filled Teeth; CAST, Caries Assessment Spectrum and Treatment Index; PI, Plaque Index; MGI, Modified Gingival Index

**Fig. 4:**
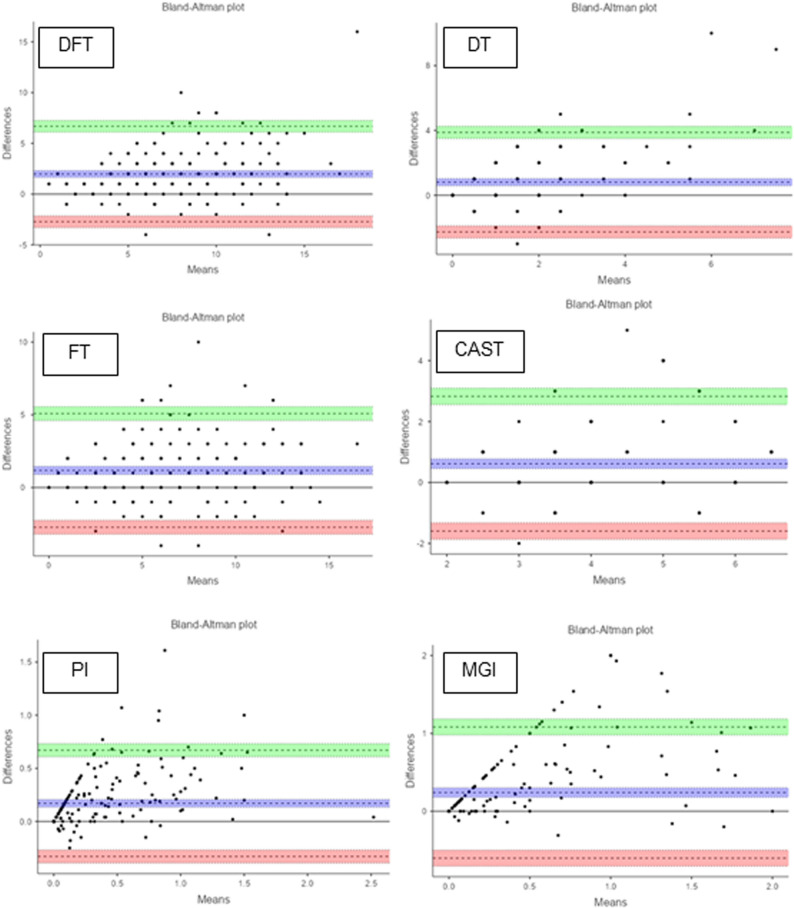
Bland-Altman plots for index measurements comparing Samsung and clinical examination (**a**) DFT Index; (**b**) DT Index; (**c**) FT Index; (**d**) CAST Index; (**e**) PI; (**f**) MGI. DFT, Decayed, Filled Teeth; DT, Decayed Teeth; FT, Filled Teeth; CAST, Caries Assessment Spectrum and Treatment Index; PI, Plaque Index; MGI, Modified Gingival Index

**Fig. 5:**
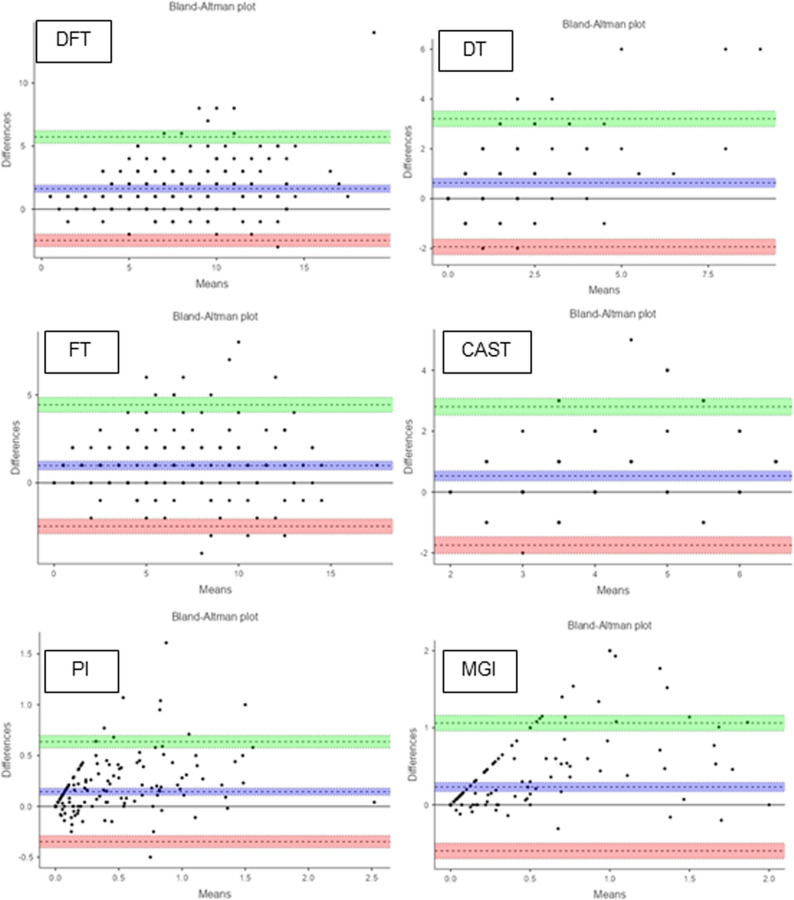
Bland-Altman plots for index measurements comparing macro camera and clinical examination (**a**) DFT Index; (**b**) DT Index; (**c**) FT Index; (**d**) CAST Index; (**e**) PI; (**f**) MGI. DFT, Decayed, Filled Teeth; DT, Decayed Teeth; FT, Filled Teeth; CAST, Caries Assessment Spectrum and Treatment Index; PI, Plaque Index; MGI, Modified Gingival Index

The agreement between photographs obtained using iPhone, Samsung, macro camera and clinical examination data was evaluated using Bland-Altman analyses (Figs. 3, 4 and 5). Measurements for DFT, DT, FT, CAST, PI, and MGI parameters across all devices generally fell within the 95% confidence intervals, indicating acceptable concordance between the two methods.

The differences between methods, as indicated on the vertical axis, were smaller for macro camera measurements than for other imaging groups. Similarly, Samsung photographs demonstrated better agreement than iPhone intraoral photographs (Figs. 3 and 4). Interpretation of index values and graphs revealed that Bland-Altman plots comparing macro camera measurements with the gold standard (clinical examination) showed data points closer to the zero line compared to Samsung and iPhone methods (Fig. 5).

Comparable agreement was observed for DT and FT scores related to decayed and filled teeth. Macro camera photographs enabled more accurate preliminary diagnoses of carious teeth. However, in patients with high caries risk, reduced consistency between measurements was noted, potentially leading to misinterpretations in photographic assessments. For CAST scores, macro camera measurements were the closest to clinical examination results. However, greater variability was observed for scores between 4 and 6, indicating inconsistencies in assessing caries depth.

## Discussion

The present study aimed to evaluate the effectiveness of clinical visual examination, recognized as the gold standard, in comparison with digital photographs obtained using smartphones and digital cameras for oral diagnosis, based on four indices: the DFT, CAST, PI, and MGI. To the best of our knowledge, no previous research in the field of teledentistry has investigated the applicability and diagnostic performance of these four distinct indices simultaneously through different diagnostic modalities.

Teledentistry has emerged as a rapidly advancing field, propelled by the widespread adoption of digital technologies and ongoing technological innovations. It enables the seamless integration of digital tools into dental practice, thereby improving access to oral healthcare for individuals in remote or underserved areas. Additionally, teledentistry supports cost-effective and efficient treatment planning, facilitates remote consultations, and enhances collaboration among dental professionals [22, 23]. A systematic review has demonstrated that teledentistry is an effective modality for caries diagnosis across various populations and dentition stages, with DMFT scores yielding results comparable to those obtained through conventional diagnostic methods. These findings highlight teledentistry’s potential as a viable alternative to traditional clinical approaches [17]. Most teledentistry studies have utilized the DMFT index for caries detection and treatment planning [24, 25]. In a 2022 study by Al-Shaya et al., digital photographs obtained via smartphones from children aged 5–10 years were compared with clinical examinations, revealing high concordance and confirming the effectiveness of teledentistry for caries detection using the DMFT index [24]. Another study comparing intraoral camera assessments with clinical examinations reported a strong positive correlation for DMFT scores [25].

The higher agreement observed in DFT scores obtained from macro camera images may be attributed to the superior image quality, resolution, and enhanced illumination provided by professional photographic systems, which facilitate clearer visualization of cavitated lesions and restorations. This is consistent with previous studies reporting that image quality plays a critical role in the diagnostic accuracy of photographic methods [7]. In contrast, although smartphone-based imaging demonstrated acceptable agreement with clinical findings, variations in lighting conditions, focus, and image sharpness may influence diagnostic consistency, as reported in smartphone-based teledentistry studies Estai et al. 2017 [12]. The relatively better performance of the Samsung device compared to the iPhone may be related to differences in camera specifications and image processing, which can affect diagnostic outcomes in digital assessments. Overall, these findings support previous evidence suggesting that teledentistry is a feasible approach for caries detection, although technical limitations of imaging devices may still influence diagnostic precision [17].

When the graphs representing DT and FT scores were analyzed, it was observed that most values remained within acceptable ranges. However, across all three methods, greater deviations were noted in measurements associated with higher mean values. These deviations suggest that the consistency of measurements tends to decrease in patients with higher caries risk. Consequently, photographic evaluations in such cases are more prone to incorrect interpretations.

Guo et al. compared macro lens photography with clinical examinations (gold standard) and, similar to our findings, observed positive correlations, with greater variability in patients with high caries prevalence [19]. Estai et al. used smartphones and assessed agreement with kappa analysis, reporting near-perfect concordance (Kappa = 0.72–0.87) [15].

Similarly, Purohit et al. evaluated DMFT scores from smartphone and clinical examinations (gold standard) using Bland-Altman analysis. Consistent with our study, most data points fell within acceptable limits, indicating agreement between methods and supporting teledentistry as a viable examination approach [26]. The lower variability in their Bland-Altman analysis compared to our study may be attributed to their focus on pediatric patients, where fewer teeth and smaller dental arches facilitate more consistent photographic assessments. In Turkey, Avcı et al. reported high agreement (Kappa = 0.83–0.87) between smartphone-based evaluations and clinical examinations [27]. The CAST index allows for the inclusion of initial carious lesions and fissure sealants without cavitation, and it evaluates dentinal lesions in two separate stages. Furthermore, it enables the recording of whether caries has reached the pulp, as well as the presence of abscesses or fistulas [18]. An additional advantage of the CAST system is that it does not require the drying of the tooth surface, which enhances its practicality and usability in various clinical and field conditions [28].

In a study by Bagińska et al., occlusal caries in the permanent first molars of children aged 6–7 years were assessed using both the DMFT and CAST indices, and the correlation between the two was analyzed. While the caries prevalence was calculated as 13.3% using the DMFT index, the prevalence of carious teeth based on CAST codes 3–6 was found to be 29.5%. This finding demonstrates that the CAST index allows for a more comprehensive recording of caries status, thereby reinforcing its value in epidemiological studies [29].

M. Aly et al. suggested that CAST-based scoring facilitated easier treatment planning in collaboration with consulting dentists and allowed for more practical monitoring of pediatric patients over time. The study emphasized the potential for more cost-effective and efficient treatment delivery using this approach [30]. The values of CAST scores most closely aligned with those obtained through clinical examination were derived from images taken with a macro camera in our study. Upon examination of the corresponding graphs, the greatest variability and deviations in CAST measurements were observed within the score range of 4–6. This increased variability in mid-range CAST scores, which reflect deeper stages of dentinal caries, may be attributed to inconsistencies in depth perception when carious lesions are evaluated via photographic images rather than direct clinical examination.

Guo et al. evaluated gingival health using the PI and MGI, comparing clinical examinations with photographs taken using a macro camera to assess the diagnostic concordance of teledentistry applications. Consistent with the present study, the agreement levels for PI and MGI were found to be lower than those for DMFT scores. This discrepancy is likely attributable to the challenges in accurately assessing subtle changes in gingival tissues—such as inflammation and bleeding—without periodontal probing, a limitation inherent to photographic evaluations but crucial for the reliability of periodontal indices [19].

Similarly, in a 2022 study conducted by Bleiel et al., smartphone photographs were utilized to assess oral health status and prosthetic treatment needs among elderly residents in nursing homes, with a mean participant age of 85.6 years. Supporting the findings of our study, the authors reported a good level of agreement for the Modified Plaque Index, underscoring the practical value of teledentistry in monitoring oral hygiene parameters, facilitating communication, and enabling remote consultation in geriatric populations [31]. This study has several limitations that should be acknowledged. Firstly, the Bland–Altman analysis employed in our study is inherently subjective, as its graphical interpretation is open to individual assessment, potentially leading to relative rather than absolute conclusions. Secondly, in the CAST index, a score of “7” indicates the presence of a pulp-related abscess or fistula. While such conditions can be diagnosed through radiographic evaluation during clinical examination, they cannot be accurately assessed through photographic analysis alone. This limitation contributes to discrepancies observed between clinical and photographic CAST scores. Another notable limitation involves the difficulty in adequately illuminating posterior teeth when using smartphone cameras. This often results in shadowed areas within the images, which may lead to an overestimation of caries depth. To overcome this challenge, the integration of auxiliary equipment—such as macro lenses and built-in lighting systems designed for smartphones, which are becoming increasingly accessible—may enhance image quality and diagnostic accuracy in future applications of teledentistry.

## Conclusion

Within the limitations of this study, the diagnostic performance of intraoral photographs obtained using two smartphones (iPhone 14 Pro and Samsung Galaxy S23 Ultra) and a digital macro camera (Canon EOS 400D) was comparable to that of clinical visual examination for the detection of DFT. These findings support the use of smartphone-based intraoral photography as a practical tool for caries assessment in teledentistry.

However, lower levels of agreement were observed for periodontal parameters, including the PI and MGI, indicating limitations of photographic methods in the evaluation of gingival conditions. Among the evaluated devices, the macro camera provided the most consistent results, followed by the Samsung smartphone, while the iPhone demonstrated relatively lower agreement with clinical findings.

Overall, intraoral digital photography represents a promising and accessible approach for remote oral health assessment, particularly for caries detection, although further improvements are needed for reliable periodontal evaluation.

## Data Availability

The datasets generated and/or analyzed during the current study are not publicly available considering that we have not required consents to publish this data, but are available from the corresponding author on reasonable request.
